# Cross-sectional study of pharmacovigilance knowledge, attitudes, and practices based on structural equation modeling and network analysis: a case study of healthcare personnel and the public in Yunnan Province

**DOI:** 10.3389/fpubh.2024.1358117

**Published:** 2024-03-19

**Authors:** Dan Qin, Fan Li, Jian Yang

**Affiliations:** ^1^School of Pharmaceutical Sciences and Yunnan Provincial Key Laboratory of Pharmacology for Natural Products, Kunming Medical University, Kunming, Yunnan, China; ^2^Yunnan Provincial Center for Drug Policy Research, Kunming, Yunnan, China; ^3^College of Modern Biomedical Industry, Kunming Medical University, Kunming, Yunnan, China; ^4^Incubation Center of Scientific and Technological Achievements, Kunming Medical University, Kunming, Yunnan, China

**Keywords:** pharmacovigilance, adverse drug reactions, knowledge attitudes and practices (KAP), healthcare personnel, public, structural equation modeling, network analysis

## Abstract

**Background:**

This study focuses on understanding pharmacovigilance knowledge, attitudes, and practices (KAP) in Yunnan Province, employing Structural Equation Modeling (SEM) and network analysis. It aims to evaluate the interplay of these factors among healthcare personnel and the public, assessing the impact of demographic characteristics to inform policy and educational initiatives.

**Methods:**

A cross-sectional survey was conducted in Yunnan, targeting healthcare personnel and the public. Data collection was through questionnaires, with subsequent analysis involving correlation matrices, network visualization, and SEM. The data analysis utilized SPSS 27.0, AMOS 26.0, and Gephi software for network analysis.

**Results:**

This study evaluated pharmacovigilance KAP among 209 public participants and 823 healthcare personnel, uncovering significant differences. Public respondents scored averages of 4.62 ± 2.70 in knowledge, 31.99 ± 4.72 in attitudes, and 12.07 ± 4.96 in practices, while healthcare personnel scored 4.38 ± 3.06, 27.95 ± 3.34, and 7.75 ± 2.77, respectively. Statistically significant correlations across KAP elements were observed in both groups, highlighting the interconnectedness of these factors. Demographic influences were more pronounced among healthcare personnel, emphasizing the role of professional background in pharmacovigilance competency. Network analysis identified knowledge as a key influencer within the pharmacovigilance KAP network, suggesting targeted education as a vital strategy for enhancing pharmacovigilance engagement.

**Conclusion:**

The research reveals a less-than-ideal state of pharmacovigilance KAP among both healthcare personnel and the public in Yunnan, with significant differences between the two groups. SEM and network analysis confirmed a strong positive link among KAP components, moderated by demographics like age, occupation, and education level. These insights emphasize the need to enhance pharmacovigilance education and awareness, thereby promoting safer drug use.

## 1 Introduction

Pharmacovigilance is crucial for drug safety and public health, particularly in emerging economies like China, where healthcare transformations necessitate improved pharmacovigilance knowledge, attitudes, and practices (KAP) among healthcare professionals and the public (1, 2). As the global pharmaceutical market evolves, countries are enhancing their pharmacovigilance systems to meet international safety standards and regulatory benchmarks, such as the Good Regulatory Practices (GRP) introduced by the World Health Organization in 2021, aiming to standardize drug safety measures worldwide (3, 4). This effort underscores pharmacovigilance’s key role in ensuring drug safety, reflecting a global consensus on the need for stringent regulatory frameworks.

The global expansion of the pharmaceutical sector highlights pharmacovigilance’s essential function in reducing adverse drug reactions (ADRs), with research showing a direct correlation between improved pharmacovigilance awareness and ADR mitigation (5, 6). However, the effectiveness of pharmacovigilance initiatives varies due to socio-economic and geographical disparities, highlighting the importance of context-specific strategies to enhance pharmacovigilance KAP. Addressing these challenges is critical for maximizing the impact of pharmacovigilance systems on drug safety and public health outcomes worldwide (7, 8). This approach is vital for leveraging pharmacovigilance to its full potential in safeguarding public health and ensuring drug safety across diverse global contexts.

SEM is a comprehensive statistical technique used for testing and estimating causal relationships using a combination of statistical data and qualitative causal assumptions. This methodology enables researchers to assess multiple and complex variables interactions simultaneously, providing a nuanced understanding of the relationships among observed and latent variables. SEM is particularly valuable in fields such as social sciences, psychology, and health sciences, where understanding the dynamics between various factors is crucial (9). By incorporating SEM in our study, we aim to delineate the intricate web of influences between KAP towards pharmacovigilance among healthcare personnel and the public in Yunnan Province. This approach allows for a more detailed analysis of how these components interact and influence each other, thus offering insights into potential leverage points for interventions.

Confirmatory factor analysis (CFA) is a powerful statistical method used to test the hypothesis that a relationship between observed variables and their underlying latent constructs exists. Unlike exploratory factor analysis (EFA), which is used to identify potential underlying structures, CFA allows researchers to test theories and hypotheses about these structures *a priori* (10). In the context of our study, CFA is employed to validate the measurement model of pharmacovigilance KAP among participants. By confirming that our observed data fit the theoretical model, CFA helps ensure the reliability and validity of the constructs being measured. This step is crucial for the subsequent SEM analysis, as it ensures that the relationships we explore are based on sound and validated measures, thereby strengthening the findings of our study.

Despite considerable research, there’s a notable gap in literature comparing pharmacovigilance KAP between healthcare professionals and the public within similar demographic settings, particularly in emerging areas like Yunnan (11). Addressing this gap is crucial, given the increasing ADR events in China and the need for effective pharmacovigilance mechanisms (12). SEM is instrumental in analyzing complex relationships among variables, making it ideal for examining pharmacovigilance KAP in Yunnan Province (13). Network analysis further aids in understanding the intricate interplay between KAP components (14).

This study aims to utilize structural equation modeling (SEM) and network analysis to elucidate pharmacovigilance KAP across diverse demographics, with the ultimate goal of enhancing global pharmacovigilance. Recognizing the global interconnectedness of health security, it seeks to highlight the significant ripple effect that regional improvements in pharmacovigilance can have on worldwide drug safety. Through a comprehensive examination of variations in pharmacovigilance KAP scores among different demographic groups and an analysis of the underlying relationships, this research intends to pinpoint specific areas that require enhancement. Furthermore, it delves into the impact of demographic characteristics on pharmacovigilance KAP, aiming to extract actionable insights that can guide the development of targeted policies and educational programs. This approach not only addresses the challenges of pharmacovigilance at both global and regional levels but also proposes strategies with the potential for worldwide application, extending well beyond the specific context of Yunnan, China. Consequently, this effort is poised to significantly contribute to the discourse on global pharmacovigilance, paving the way for improved drug safety across various populations.

## 2 Methods

### 2.1 Research design and methods

#### 2.1.1 Sampling strategy and size calculation

A cross-sectional survey was conducted across Yunnan Province, from January 15 to March 30, 2023, utilizing a strategic and two-tiered sampling approach to ensure the collection of a comprehensive and representative dataset. For healthcare professionals, a stratified random sampling technique was rigorously applied across medical institutions of various levels. This method was designed to minimize selection bias and enhance the sample’s representativeness, ensuring extensive coverage across a wide range of demographic and professional backgrounds. This deliberate strategy aimed to encompass a diverse array of healthcare professionals, aligning with the objective to generate insights with high external validity. In contrast, the public sample was obtained through convenience sampling, chosen for its practicality and efficiency in data collection. This method was recognized for the potential bias it might introduce, yet it was deemed suitable for the exploratory nature of assessing public pharmacovigilance knowledge.

A calculated sample size underpinned the robust methodology, ensuring the statistical significance and reliability of the findings. The sample size for healthcare professionals was determined using the formula: *n = Z^2^ × p × (1 − p)/E^2^*, where ‘*Z*’ represents the *Z-value* corresponding to a 95% confidence level (1.96), ‘*p*’ is the estimated proportion of healthcare professionals with adequate pharmacovigilance knowledge (conservatively set at 0.5 based on extensive literature review and preliminary research), and ‘*E*’ refers to the margin of error. The choice of ‘*p*’ at 0.5 reflects a conservative yet informed estimate, aiming to balance the expected diversity in pharmacovigilance knowledge levels among professionals. An ‘*E*’ value of 0.05 was selected for healthcare professionals to achieve precise estimates critical for assessing the impact on pharmacovigilance outcomes.

The calculated sample size for healthcare professionals is approximately 384, derived as follows: *n = 1.96^2^ × 0.5 × (1–0.5)/0.05^2^ = 384.16.* For the general public, a higher margin of error (‘*E*’ value of 0.1) was accepted to reflect the broader variability expected and the exploratory nature of the public knowledge assessment. This adjustment resulted in a theoretical sample size of approximately 96 individuals: *n = 1.96^2^ × 0.5 × (1–0.5)/0.1^2^ = 96.04.*

This dual sampling approach, combining stratified random sampling for healthcare professionals with convenience sampling for the public, was specifically designed to balance scientific rigor with practical feasibility. The differential ‘*E*’ values for healthcare professionals and the general public were deliberately chosen to reflect the distinct objectives and expected variability within each group, ensuring tailored analysis and interpretation of the collected data.

#### 2.1.2 Methodology: questionnaire design and validation

The questionnaire, integral for assessing pharmacovigilance KAP in this study, was developed in consultation with pharmacovigilance experts and underwent a validation process via a pilot study to ensure its reliability and alignment with the study’s aims. For the general population, it featured multiple-choice questions divided into three parts: a knowledge section consisting of five questions (totaling up to 8 points) where K4 is scored based on option level and other questions are scored as 1 point for correct and 0 points for incorrect or unknown answers; an attitudes section with seven 5-level Likert scale questions (totaling up to 35 points), scored from “strongly disagree” (1 point) to “strongly agree” (5 points); and a practices section comprising seven questions scored based on option level, with a total of 16 points. For healthcare professionals, the questionnaire, tailored according to the “Clinical Medication Management Standards for Medical Institutions” by the National Health Commission of China, included five multiple-choice knowledge questions (1 point for correct, 0 points for incorrect or unknown answers, totaling 5 points), six 5-level Likert scale attitude questions (similar to the public section, totaling 30 points), and five practice questions scored based on option level, with a total of 10 points. This detailed construction ensures a comprehensive evaluation of pharmacovigilance KAP across diverse demographics, enhancing the study’s methodological transparency and contributing to the field’s body of knowledge (The questionnaire is appended to the manuscript as a Supplementary files, enhancing methodological transparency and allowing detailed scrutiny of the instruments used).

#### 2.1.3 Data collection and processing

This study’s quantitative cross-sectional approach meticulously explores pharmacovigilance KAP among Yunnan Province’s healthcare personnel and the general population. Targeting those 25+ in healthcare and 18+ in the general population, it necessitates normal cognitive abilities for all, with a prerequisite of 1 year’s medical field experience for professionals. Selection from five distinct Yunnan regions ensured a dataset representative of diverse pharmacovigilance perspectives. Ethical adherence was critical, starting with informed consent to reinforce the study’s integrity and rigor. Surveys, distributed via email using Likert scale questions, assessed pharmacovigilance KAP, pinpointing challenges and improvement opportunities. Online questionnaires facilitated data gathering, processed with SPSS 27.0, encompassing data cleaning, exploratory analysis (EDA), and validation of SEM analysis’s essential assumptions. This methodology underscores our commitment to deriving meaningful insights into Yunnan’s pharmacovigilance landscape, contributing valuable knowledge to the field.

#### 2.1.4 Construction and analysis of the structural equation model

The theory of KAP is frequently utilized in health and public health interventions, positing that knowledge forms the basis for developing positive and accurate attitudes, which in turn, influence behavioral changes. This study, drawing on the KAP framework, posits a series of hypotheses concerning the interrelations among pharmacovigilance KAP. It involves a comprehensive procedure of reliability and validity analysis, including CFA and internal consistency assessment of the survey data. Utilizing AMOS 26.0 software, a structural equation model (SEM) was constructed to evaluate the model’s Fit Index, test the path coefficients, analyze the interrelations between pharmacovigilance KAP, and assess the impact of demographic characteristics on these variables.

To elucidate, the study proposes the following hypotheses based on the KAP theory and demographic characteristics: Hypothesis 1 (*H1* and *H1*Δ): Asserts that pharmacovigilance knowledge positively influences attitudes. Hypothesis 2 (*H2* and *H2*Δ): Suggests that pharmacovigilance attitudes positively impact practices. Hypothesis 3 (*H3* and *H3*Δ): Indicates that pharmacovigilance knowledge directly contributes to improved practices. Hypothesis 4 (*H4* and *H4*Δ): Posits that demographic characteristics enhance pharmacovigilance knowledge. Hypothesis 5 (*H5* and *H5*Δ): Advocates that demographic features positively affect pharmacovigilance attitudes. Hypothesis 6 (*H6* and *H6*Δ): Proposes that demographic variables positively influence pharmacovigilance practices (“Δ” to denote indirect effects mediated by demographic characteristics, offering a more nuanced understanding of how these variables interact within the KAP framework).

### 2.2 Network analysis

In the exploration of pharmacovigilance KAP within Yunnan Province, advanced network analysis was conducted using Gephi software, transforming collected data into an adjacency matrix. This process enabled the construction of a detailed network graph, where nodes represented the various KAP elements and edges depicted the connections between these elements. The focus was placed on centrality and modularity metrics to identify the most influential nodes and to uncover distinct clusters within the network. This analysis provided critical insights into the structural dynamics of pharmacovigilance among different demographic groups of healthcare personnel and the public. By examining these interconnections, key areas for targeted interventions and policy development in the field of pharmacovigilance were identified, enhancing the collective understanding of the complex relationships within KAP.

### 2.3 Statistical analysis

To rigorously examine pharmacovigilance KAP among healthcare professionals and the general public in Yunnan Province, this investigation employed SPSS 27.0 and AMOS 26.0 for extensive statistical analyses. Descriptive statistics illuminated the demographic and professional profiles of the participants, laying the groundwork for further analysis. The study’s KAP measures demonstrated high reliability, as evidenced by Cronbach’s alpha values of 0.925 for the public and 0.803 for healthcare professionals, indicating robust internal consistency. EFA uncovered the latent dimensions of pharmacovigilance engagement, while CFA validated these dimensions against the theoretical framework. SEM revealed complex relationships between KAP factors and demographic variables, highlighting both direct and indirect influences on pharmacovigilance behaviors. Adhering to a significance level of α = 0.05, these analyses not only affirmed the statistical integrity of the findings but also enriched the understanding of pharmacovigilance KAP in Yunnan Province. Additionally, the Kaiser-Meyer-Olkin (KMO) measure confirmed sampling adequacy with values of 0.925 and 0.863, further ensuring the methodological soundness of the study.

## 3 Results

### 3.1 Basic information

#### 3.1.1 Public participants

In this study, a total of 211 questionnaires were distributed among the general public, with 209 responses deemed valid, achieving a 99.1% response rate. The demographic distribution revealed a predominance of females (70.3%) over males (29.7%), with a significant portion aged 18–30 years (54.1%). Key occupational groups included healthcare professionals (42.6%), students (23.4%), and public officials/employees (12.4%), with 15.8% reporting chronic diseases. For public participants, the average scores for pharmacovigilance knowledge, attitude, and practice were 4.62 ± 2.70, 31.99 ± 4.72, and 12.07 ± 4.96, respectively, translating to awareness, holding, and execution rates of 57.75, 91.40, and 75.44%.

#### 3.1.2 Healthcare personnel

The survey engaged 823 participants from the healthcare sector, predominantly females (81.7%) and most within the 31–50 age range (55.7%). In terms of education, a significant portion (66.8%) reported having bachelor’s degrees, and a vast majority (96.4%) had obtained a college degree or higher. This professional group was composed of doctors (29.0%), pharmacists (34.0%), and nurses (26.9%), with 34.9% holding positions at the teacher/assistant level. The affiliations of these healthcare professionals varied, including township health centers (28.6%), community health service centers (24.2%), tertiary comprehensive hospitals (22.6%), and secondary comprehensive hospitals (18.8%). The pharmacovigilance knowledge, attitude, and practice scores for healthcare professionals averaged 4.38 ± 3.06, 27.95 ± 3.34, and 7.75 ± 2.77, respectively, reflecting awareness, holding, and execution rates of 87.6, 93.17, and 77.5%.

### 3.2 Structural equation model construction and fitting results

#### 3.2.1 Construction of the public pharmacovigilance KAP model

**Figure 1:**
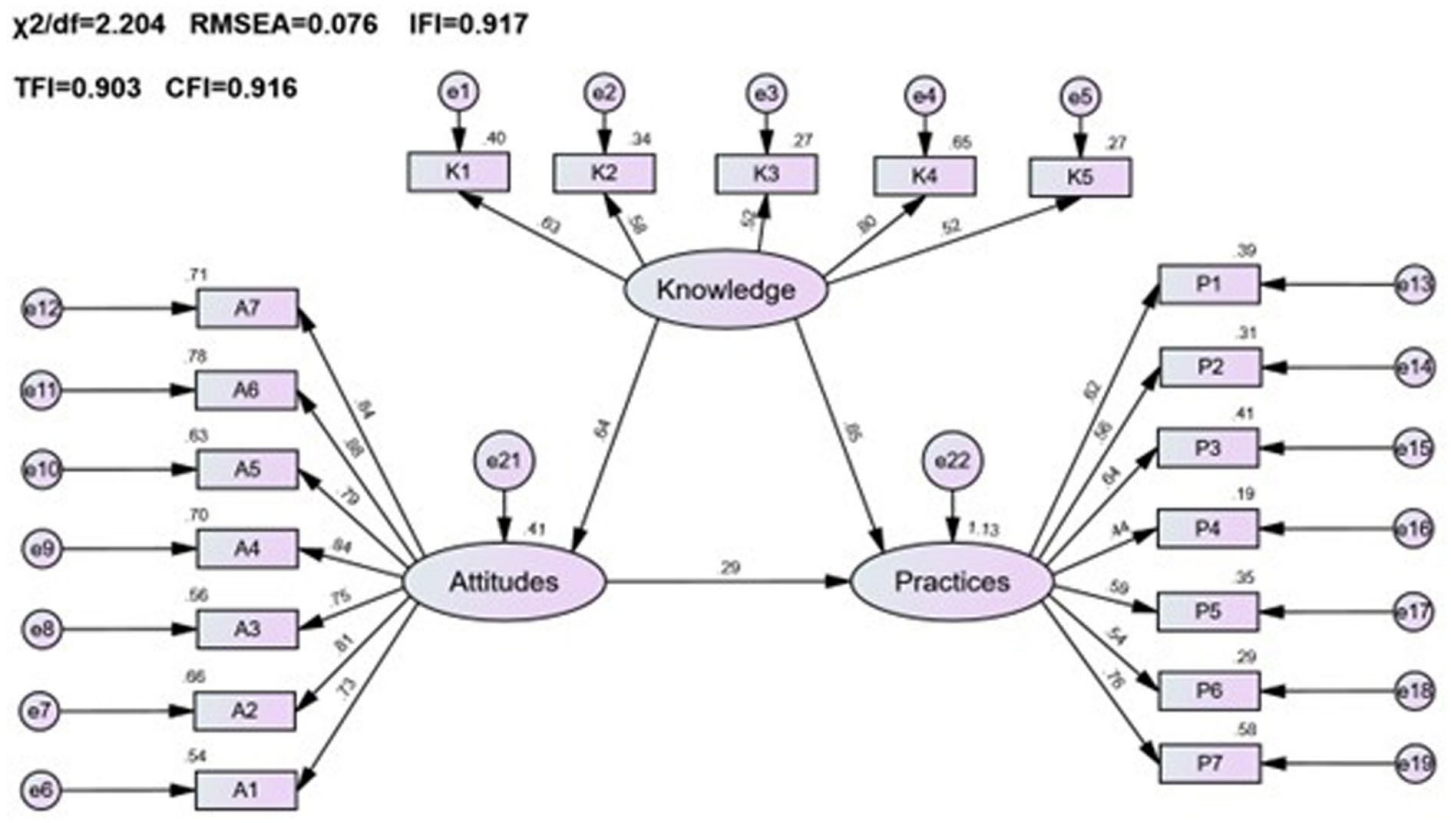
Fit results of the SEM for the general public’s pharmacovigilance KAP.

In this study, a public pharmacovigilance KAP model was constructed using factor analysis for validation, ensuring robust and reliable results. The standardized loadings for the public KAP factors (see Table 1) ranged between 0.4 and 0.9 (*p* < 0.001), indicating statistically significant findings. The average variance extracted (AVE) for pharmacovigilance knowledge and attitude were above 0.5, with composite reliability (CR) values exceeding 0.7, confirming strong measurement extractability for these factors. Despite a slightly lower AVE of 0.44 for pharmacovigilance practice, its CR stood at a solid 0.808, denoting good measurement extractability. Based on CFA outcomes and hypotheses *H1*, *H2*, and *H3*, a structured equation model representing the interplay of public pharmacovigilance knowledge, attitude, and practice was formulated and illustrated in Figure 1.

**Table 1:** Results of confirmatory factor analysis for the general public’s KAP.

Paths			Unstandardized loadings	S.E.	C.R.	*p*	Standardized loadings	AVE	CR
K1	<---	Knowledge	1				0.635	0.522	0.787
K2	<---	Knowledge	0.918	124	7.404	<0.001	0.583		
K3	<---	Knowledge	0.792	0.118	6.727	<0.001	0.521		
K4	<---	Knowledge	2.812	0.293	9.611	<0.001	0.805		
K5	<---	Knowledge	0.468	0.069	6.757	<0.001	0.523		
A1	<---	Attitude	1				0.734	0.652	0.928
A2	<---	Attitude	1.175	0.097	12.110	<0.001	0.815		
A3	<---	Attitude	1.359	0.125	10.842	<0.001	0.748		
A4	<---	Attitude	1.370	0.112	12.179	<0.001	0.837		
A5	<---	Attitude	1.347	0.118	11.403	<0.001	0.791		
A6	<---	Attitude	1.333	0.104	12.778	<0.001	0.885		
A7	<---	Attitude	1.325	0.110	12.070	<0.001	0.840		
P1	<---	Practice	1				0.621	0.44	0.808
P2	<---	Practice	1.016	0.139	7.289	<0.001	0.559		
P3	<---	Practice	1.111	0.135	8.228	<0.001	0.643		
P4	<---	Practice	0.754	0.128	5.908	<0.001	0.438		
P5	<---	Practice	0.997	0.130	7.662	<0.001	0.593		
P6	<---	Practice	0.719	0.102	7.062	<0.001	0.539		
P7	<---	Practice	2.777	0.298	9.325	<0.001	0.764		

#### 3.2.2 Fitting results of the public pharmacovigilance KAP model

The public pharmacovigilance KAP model demonstrated a good fit in this study, with fitting indices meeting or surpassing fit standards (*χ^2^*/df = 2.204, RMSEA = 0.076, IFI = 0.917, TLI = 0.906, CFI = 0.916), indicating the model’s validity (Table 2).

**Table 2:** Fit Index for the structural equation model of pharmacovigilance KAP for the general public.

Fit Index	*χ*^2^/*df*	RMSEA	IFI	TLI	CFI
Fit standards	<5.00	<0.08	>0.90	>0.90	>0.90
Fit results	2.204	0.076	0.917	0.906	0.916

RMSEA, root mean square error of approximation; IFI, Incremental Fit Index; TLI, Tucker–Lewis Index; CFI, Comparative Fit Index.

Significant positive correlations were found among the KAP elements (*p* < 0.001), as detailed in Table 3 and Figure 1. Path analysis showed that knowledge positively influenced attitude (path coefficient = 0.64) and practice (path coefficient = 0.85), while attitude impacted practice (path coefficient = 0.29).

**Table 3:** Path coefficients for the KAP model of pharmacovigilance for the general public.

Paths			Estimate	S.E.	C.R.	*p*	Label
Attitude	<---	Knowledge	0.923	0.140	6.595	***	*H1*
Practice	<---	Knowledge	0.856	0.124	6.905	***	*H3*
Practice	<---	Attitude	0.199	0.047	4.195	***	*H2*

SE, standard error; CR, critical ratio.

Further, mediation analysis (Table 4) revealed that knowledge had both a direct (0.856) and an indirect effect (0.184 through attitude) on practice, resulting in a total effect of 1.04. This highlights the complex interplay between knowledge and practice, with attitude serving as a key mediator in converting pharmacovigilance knowledge into actionable practices.

**Table 4:** Mediating effects of pharmacovigilance knowledge and practice for the general public.

Parameter	Estimate	Lower	Upper	*p*
Indirect effects	0.184	0.109	0.311	0.000
Direct effects	0.856	0.662	1.067	0.002
Total effects	1.040	0.848	1.260	0.001

#### 3.2.3 Construction of healthcare personnel pharmacovigilance KAP model

**Figure 2:**
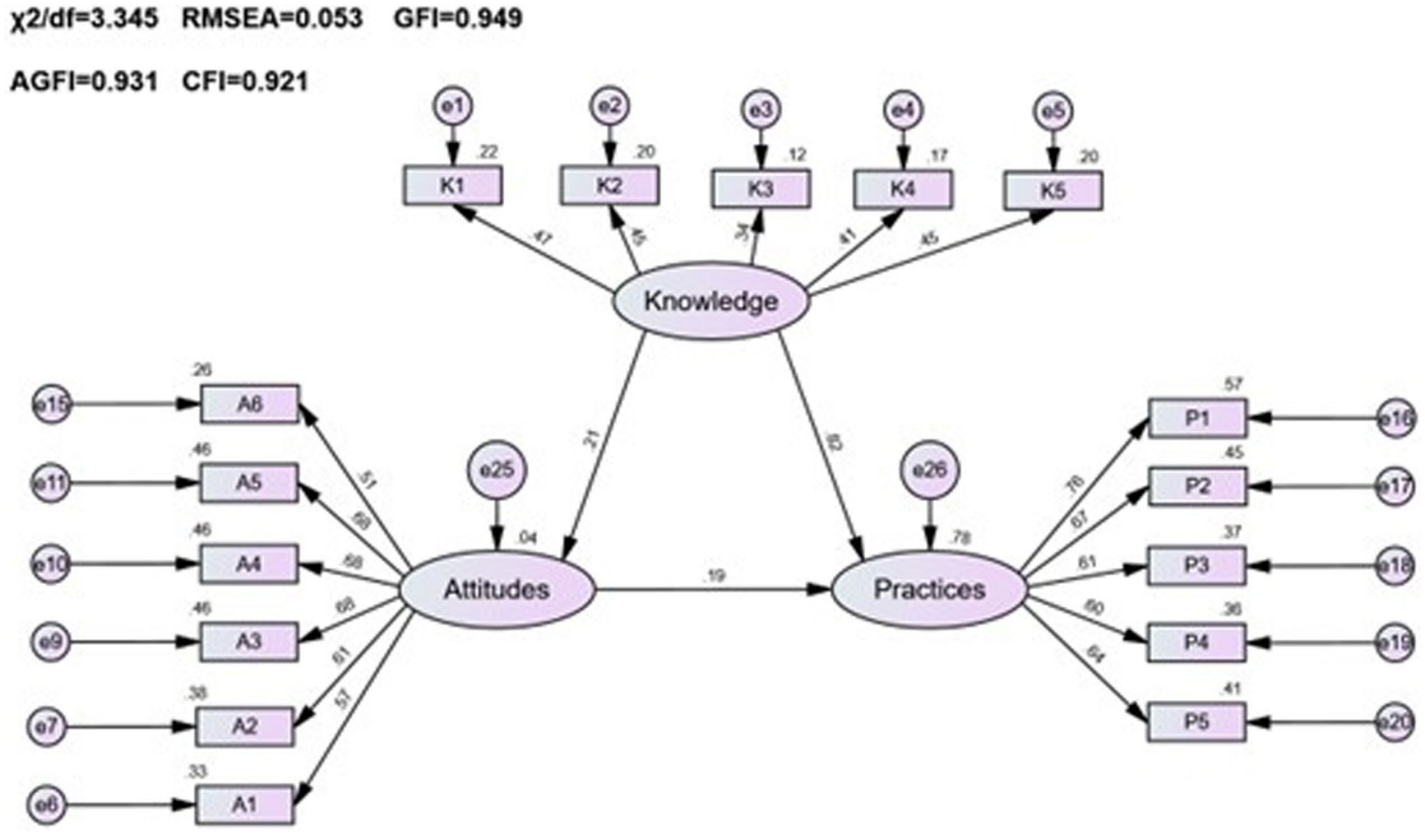
Fit results of the SEM for healthcare personnel’s pharmacovigilance KAP.

A pharmacovigilance KAP model was developed for healthcare personnel, employing factor analysis for validation. The model’s standardized loadings, as presented in Table 5, varied from 0.3 to 0.8 (*p* < 0.001), indicating statistically significant outcomes. Analysis showed that the AVE for the attitude and practice factors slightly missed the preferred mark of 0.5, yet the CR values were above 0.7, demonstrating satisfactory reliability. In contrast, the AVE for knowledge stood notably lower at 0.181 with a CR of 0.506, highlighting areas for improvement in knowledge indicator reliability. Following CFA results and hypotheses *H1*Δ, *H2*Δ, and *H3*Δ, a structural equation model was established to illustrate the interconnections among healthcare personnel’s pharmacovigilance KAP, as depicted in Figure 2.

**Table 5:** Results of confirmatory factor analysis for healthcare personnel’s KAP.

Paths			Unstandardized loadings	S.E.	C.R.	*p*	Standardized loadings	AVE	CR
K1	<---	Knowledge	1				0.465	0.181	0.506
K2	<---	Knowledge	0.878	0.104	8.404	<0.001	0.449		
K3	<---	Knowledge	0.700	0.104	6.741	<0.001	0.340		
K4	<---	Knowledge	0.375	0.047	8.047	<0.001	0.411		
K5	<---	Knowledge	0.723	0.085	8.513	<0.001	0.446		
A1	<---	Attitude	1				0.572	0.384	0.787
A2	<---	Attitude	1.397	0.106	13.169	<0.001	0.614		
A3	<---	Attitude	1.363	0.099	13.804	<0.001	0.677		
A4	<---	Attitude	1.343	0.100	13.413	<0.001	0.679		
A5	<---	Attitude	1.393	0.102	13.595	<0.001	0.681		
A6	<---	Attitude	1.202	0.109	11.069	<0.001	0.505		
P1	<---	Practice	1				0.755	0.419	0.780
P2	<---	Practice	1.044	0.059	17.556	<0.001	0.667		
P3	<---	Practice	0.839	0.051	16.372	<0.001	0.609		
P4	<---	Practice	1.147	0.077	14.932	<0.001	0.598		
P5	<---	Practice	1.200	0.075	15.965	<0.001	0.643		

SE, standard error; CR, critical ratio; AVE, average of variance extracted; CR, composite reliability.

#### 3.2.4 Fitting results of healthcare personnel pharmacovigilance KAP model

The healthcare personnel pharmacovigilance KAP model underwent a meticulous fitting process, showcasing favorable fitting indices, as substantiated by Table 6: *χ*^2^/*df* = 3.345, RMSEA = 0.053, GFI = 0.949, AGFI = 0.931, and CFI = 0.921, all of which adhered to or surpassed their respective fit standards, thus corroborating a commendable fit between the model and the data. Diving into the specifics of model fitting, as illustrated in Table 7 and Figure 2, it was discerned that knowledge significantly and positively influenced attitude, evinced by a path coefficient of 0.21. Furthermore, attitude exhibited a significant positive impact on practice, denoted by a path coefficient of 0.19. Notably, knowledge imparted a substantial positive impact on practice, with a path coefficient of 0.82. Exploring mediation effects, Table 8 unfolds that knowledge indirectly influenced practice, with a direct effect of 1.57 and an indirect effect of 0.073, channeled through attitude towards practice, accumulating to a total effect of 1.643.

**Table 6:** Fit Index for the structural equation model of pharmacovigilance knowledge, attitude, and practice for healthcare personnel.

Fit Index	*χ*^2^/*df*	RMSEA	GFI	AGFI	CFI
Fit standards	<5.00	<0.08	>0.90	>0.90	>0.90
Fit results	3.345	0.053	0.949	0.931	0.921

RMSEA, root mean square error of approximation; GFI, Goodness-of-Fit Index; AGFI, Adjusted Goodness-of-Fit Index; CFI, Comparative Fit Index.

**Table 7:** Path coefficients for the KAP model of pharmacovigilance for healthcare personnel.

Paths			Estimate	S.E.	C.R.	*p*	Label
Attitude	<---	Knowledge	0.306	0.088	3.481	***	*H1*
Practice	<---	Knowledge	1.570	0.171	9.196	***	*H3*
Practice	<---	Attitude	0.240	0.059	4.041	***	*H2*

SE, standard error; CR, critical ratio.

**Table 8:** Mediating effects of pharmacovigilance knowledge and practice for healthcare personnel.

Parameter	Estimate	Lower	Upper	*p*
Indirect effects	0.073	0.037	0.139	0.002
Direct effects	1.570	1.157	2.156	0.002
Total effects	1.643	1.233	2.222	0.002

### 3.3 Influence of demographic characteristics on pharmacovigilance KAP

#### 3.3.1 Influence of demographic characteristics on public pharmacovigilance KAP

**Figure 3:**
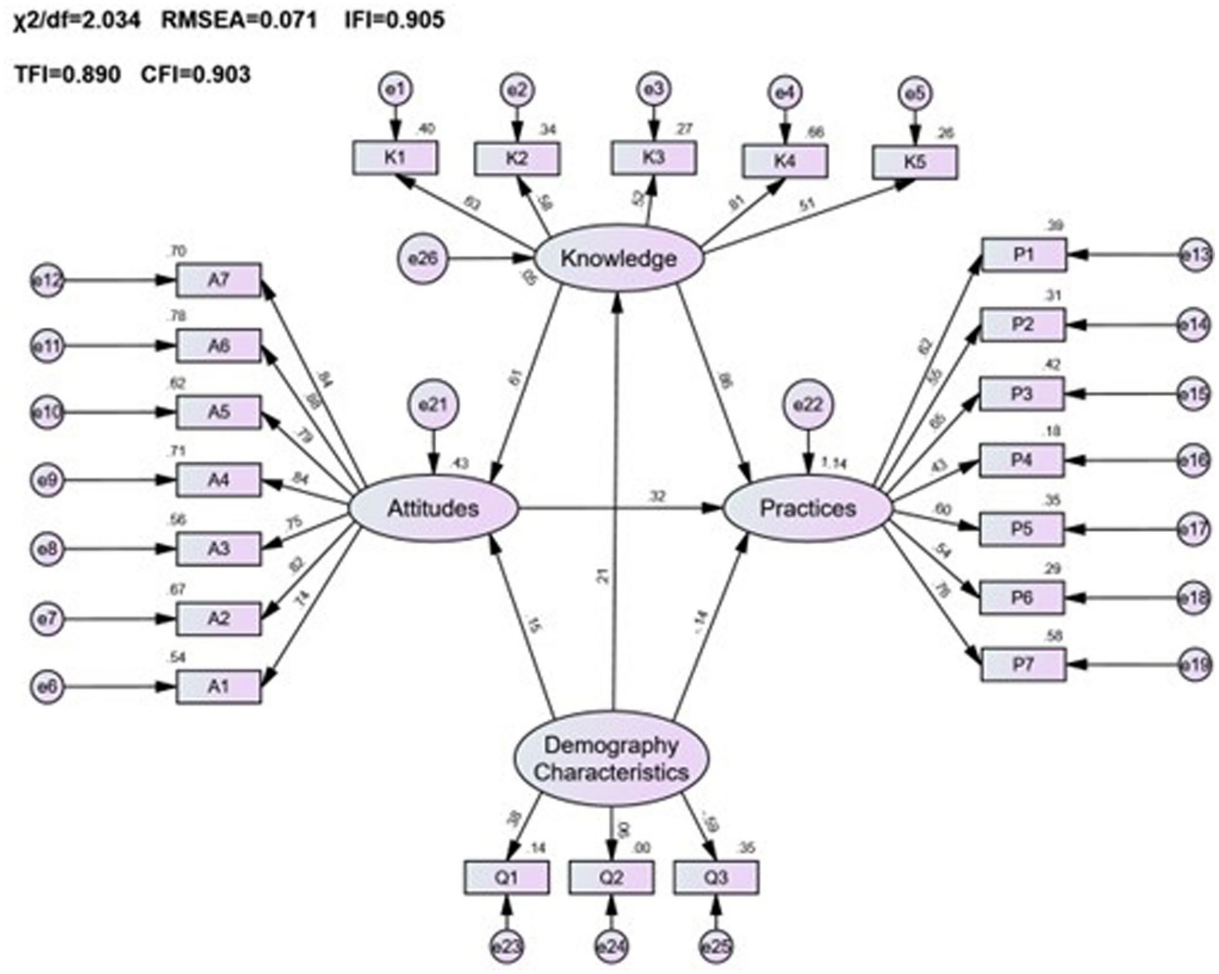
The KAP model of pharmacovigilance for the general public and its correlation with demographic characteristics.

This research sought to understand the influence of demographic characteristics on public pharmacovigilance KAP by incorporating these demographics as variables in the public KAP model. Analysis of the model’s fitting indices (*χ*^2^/*df* = 2.034, RMSEA = 0.071, IFI = 0.905, TLI = 0.890, CFI = 0.903), as shown in Table 9, confirmed a satisfactory fit, adhering to established fit criteria. Despite this, the detailed examination of the model, as represented in Figure 3 and Table 10, revealed that age, gender, and occupation were not statistically significant in influencing the KAP metrics (*p* > 0.05). This indicates a minimal direct effect of these demographic variables on public pharmacovigilance behaviors. Nonetheless, the significant paths from knowledge to attitude (0.882, *p* < 0.001) and from attitude to practice (0.226, *p* < 0.001) highlight the core interactions within the KAP model, emphasizing the integral role of knowledge and attitude in shaping pharmacovigilance practices, independent of demographic distinctions.

**Table 9:** Fit Index for the KAP model of pharmacovigilance for the general public and its correlation with demographic characteristics.

Fit Index	*χ*^2^/*df*	RMSEA	IFI	TLI	CFI
Fit standards	<5.00	<0.08	>0.90	>0.90	>0.90
Fit results	2.034	0.071	0.905	0.890	0.903

RMSEA, root mean square error of approximation; IFI, Incremental Fit Index; TLI, Tucker–Lewis Index; CFI, Comparative Fit Index.

**Table 10:** Path coefficients for the KAP model of pharmacovigilance for the general public and its correlation with demographic characteristics.

Paths			Estimate	S.E.	C.R.	*p*	Label
Knowledge	<---	Demographic characteristics	−0.251	0.171	1.467	0.143	*H5*
Attitude	<---	Knowledge	0.882	0.142	6.221	***	*H1*
Attitude	<---	Demographic characteristics	0.255	0.205	1.245	0.213	*H4*
Practice	<---	Knowledge	0.867	0.127	6.811	***	*H3*
Practice	<---	Attitude	0.226	0.051	4.446	***	*H2*
Practice	<---	Demographic characteristics	−0.169	0.095	−1.790	0.073	*H6*

SE, standard error; CR, critical ratio.

#### 3.3.2 Influence of demographic characteristics on healthcare personnel pharmacovigilance KAP

In analyzing the impact of demographic characteristics on the pharmacovigilance KAP among healthcare personnel, variables including age, gender, work experience, education level, professional title, role, and the type of institution were evaluated. The fitting indices for the model (*χ*^2^/*df* = 5.189, RMSEA = 0.071, GFI = 0.879, AGFI = 0.851, CFI = 0.809) confirmed a satisfactory alignment with the accepted standards, as detailed in Table 11.

**Table 11:** Fit Index for the KAP model of pharmacovigilance for healthcare personnel and its correlation with demographic characteristics.

Fit Index	*χ*^2^/*df*	RMSEA	GFI	AGFI	CFI
Fit standards	<5.00	<0.08	>0.90	>0.90	>0.90
Fit results	5.189	0.071	0.879	0.851	0.809

RMSEA, root mean square error of approximation; GFI, Goodness-of-Fit Index; AGFI, Adjusted Goodness-of-Fit Index; CFI, Comparative Fit Index.

**Figure 4:**
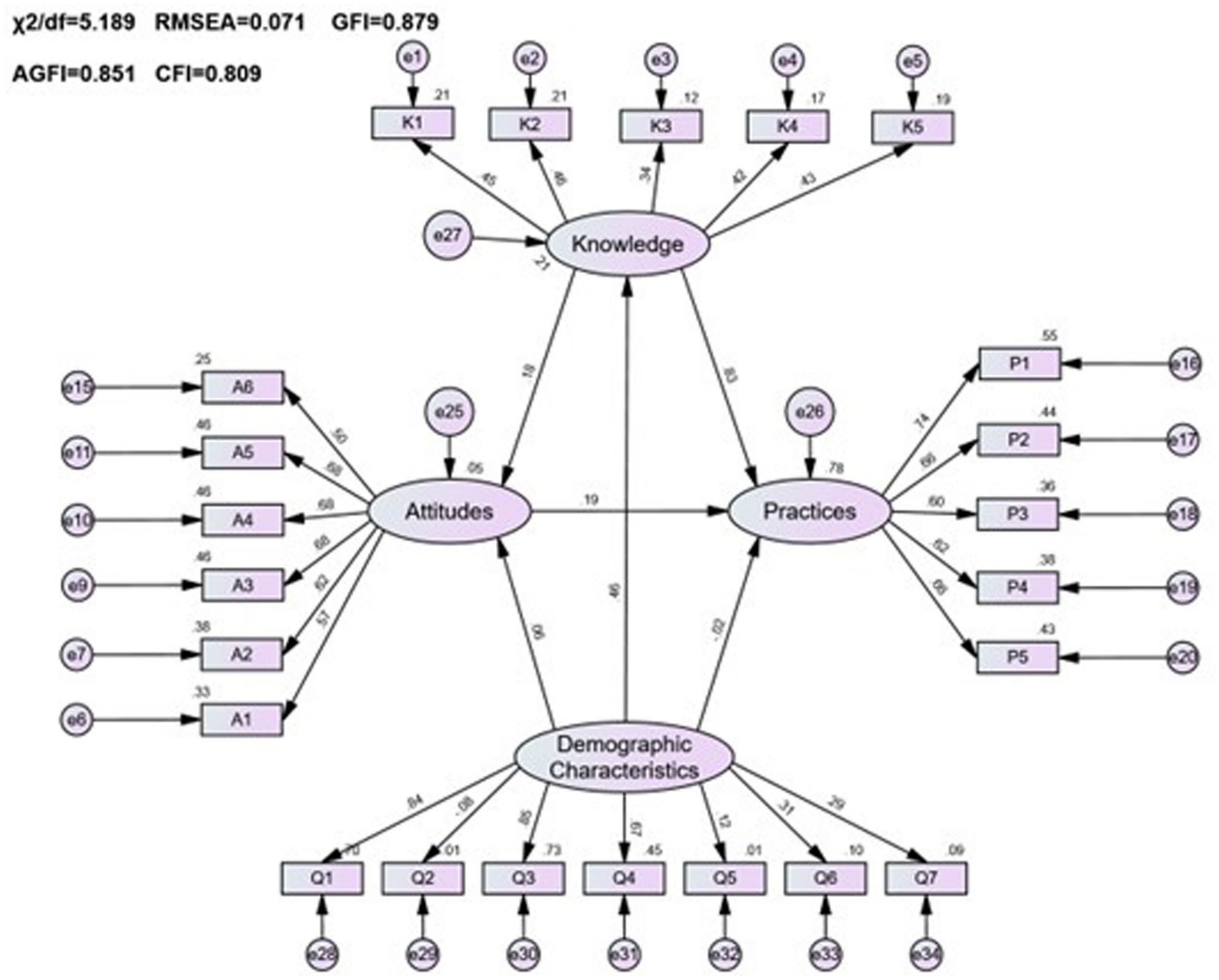
The KAP model of pharmacovigilance for healthcare personnel and its correlation with demographic characteristics.

The analysis, as depicted in Figure 4 and Table 12, revealed that demographic factors largely had a positive effect on the pharmacovigilance KAP of healthcare personnel, with the exception of gender. Key variables such as age, level of education, professional title, role, and institutional context were found to significantly contribute to enhancing pharmacovigilance KAP. Specifically, demographic variables were significant predictors of knowledge (estimate = 0.125, *p* < 0.001), while their influence on practice was minimal (estimate = −0.011, *p* = 0.708), highlighting the nuanced role demographics play in shaping pharmacovigilance engagement among healthcare personnel.

**Table 12:** Path coefficients for the KAP model of pharmacovigilance for healthcare personnel and its correlation with demographic characteristics.

Paths			Estimate	S.E.	C.R.	*p*	Label
Knowledge	<---	Demographic characteristics	0.125	0.016	7.630	***	*H5*
Attitude	<---	Knowledge	0.275	0.106	2.589	0.010	*H1*
Attitude	<---	Demographic characteristics	0.024	0.022	1.081	0.280	*H4*
Practice	<---	Knowledge	1.601	0.200	7.999	***	*H3*
Practice	<---	Attitude	0.243	0.058	4.157	***	*H2*
Practice	<---	Demographic characteristics	−0.011	0.029	−0.375	0.708	*H6*

SE, standard error; CR, critical ratio.

### 3.4 Network analysis results

Using a comprehensive dataset from a cross-sectional study involving both healthcare personnel and the public in Yunnan Province, we embarked on an intricate network analysis to decipher the relationships within the pharmacovigilance KAP framework.

#### 3.4.1 Differential network patterns in pharmacovigilance awareness: healthcare personnel versus public

**Figure 5:**
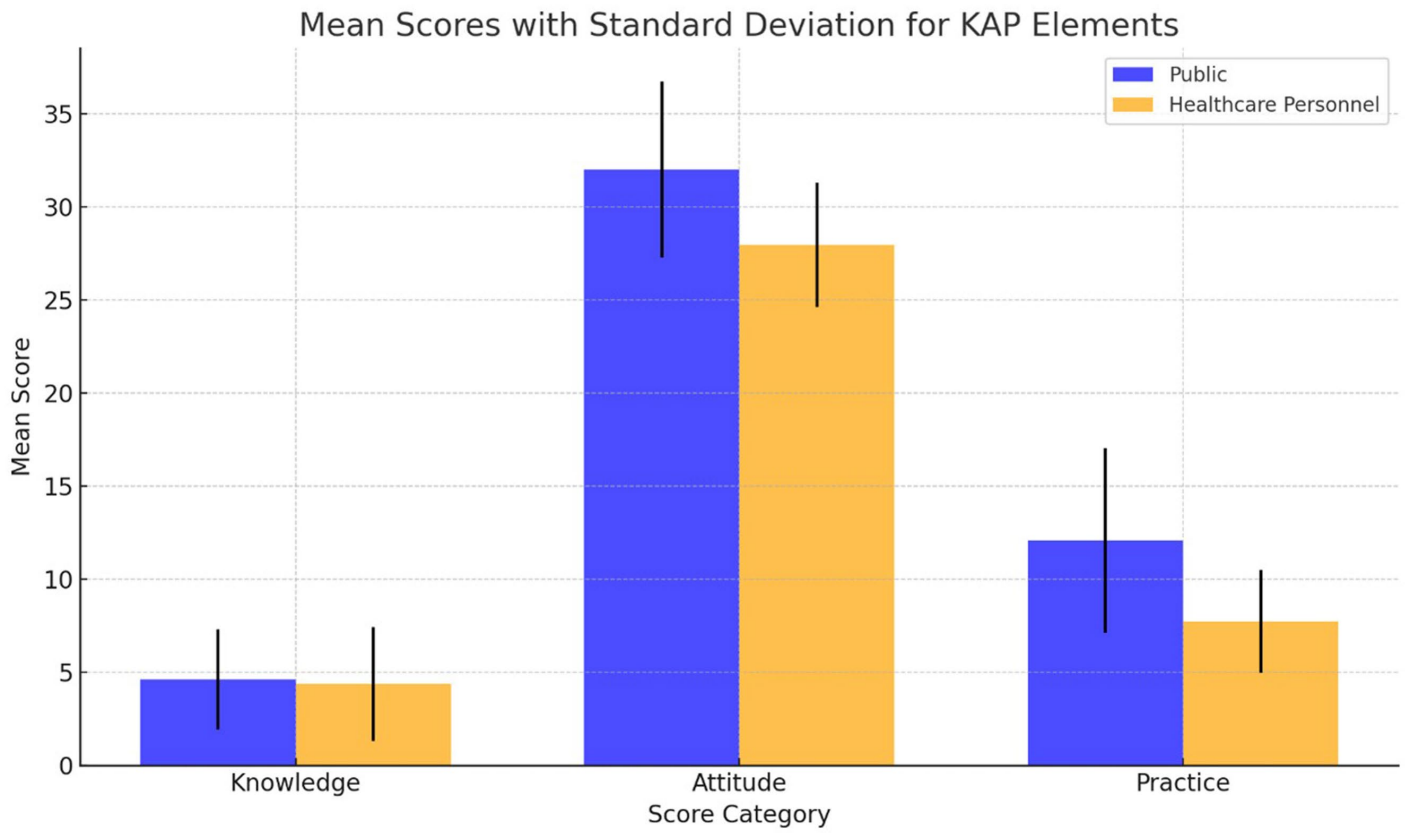
Comparative analysis of mean KAP scores among public and healthcare personnel.

Figure 5 contrasts KAP scores between the public and healthcare personnel. Contrarily to initial assumptions, the public outperforms in knowledge (4.62 ± 2.70) and attitudes (31.99 ± 4.72), while healthcare personnel excel in practices (7.75 ± 2.77) but lag in knowledge (4.38 ± 3.06). Table 13, further dissects these differences, underscoring targeted educational interventions’ importance. Network analysis elucidates key intervention areas, spotlighting the critical need to elevate pharmacovigilance understanding across both demographics to foster comprehensive awareness and application. This nuanced approach underscores divergent strengths and educational gaps in pharmacovigilance competencies.

**Table 13:** Descriptive statistics for knowledge, attitude, and practice scores.

Metric	Public knowledge	Public attitude	Public practice	Public awareness	Healthcare knowledge	Healthcare attitude	Healthcare practice
Average score	4.62	31.99	12.07	36.62	4.38	27.95	7.75
Standard deviation	2.70	4.72	4.96	5.35	3.06	3.34	2.77

#### 3.4.2 Centrality of knowledge in pharmacovigilance KAP dynamics

**Figure 6:**
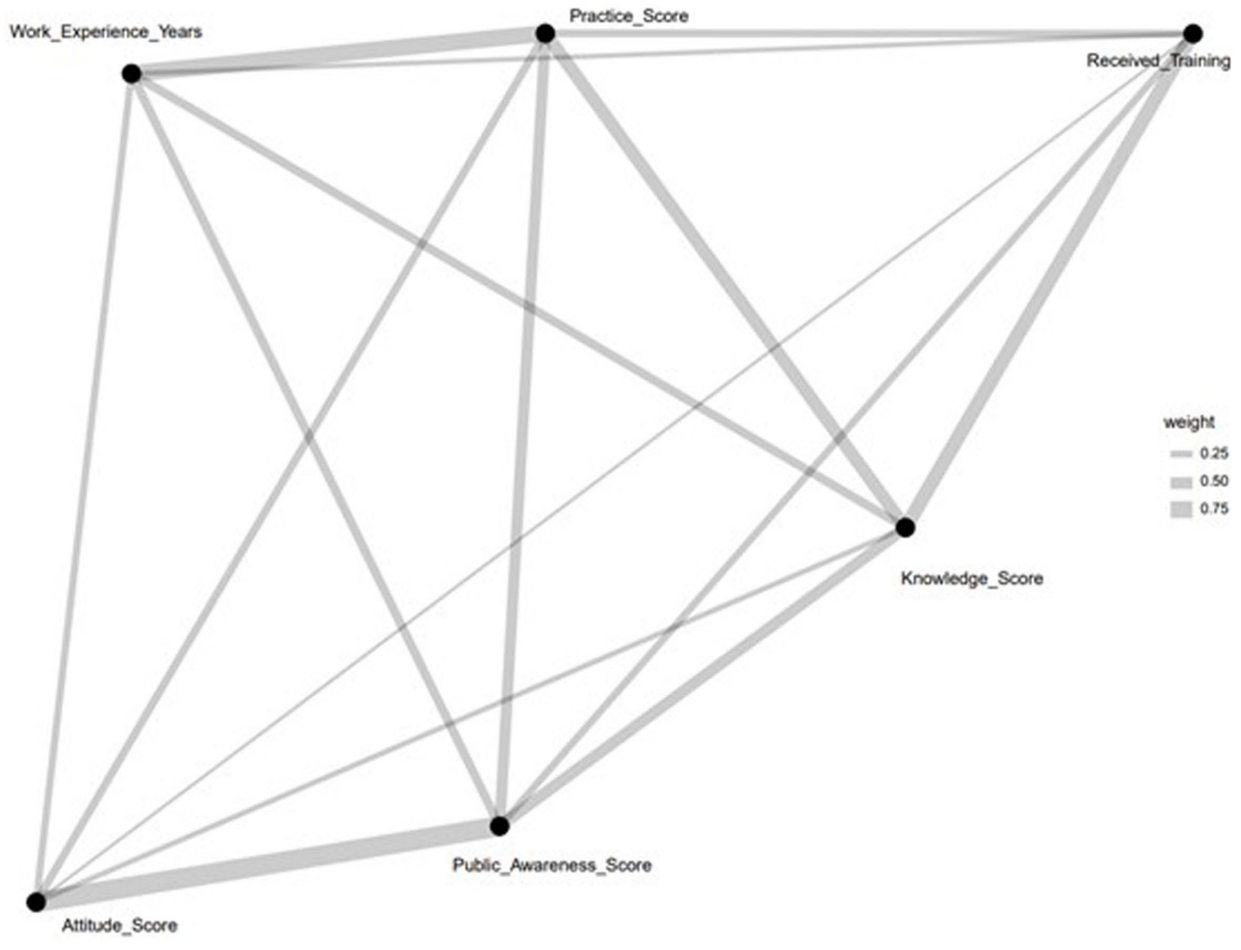
Network diagram of KAP elements.

Figure 6 presents a network diagram that maps the relationships among key pharmacovigilance Knowledge, Attitude, and Practice (KAP) variables within healthcare personnel. It identifies ‘Received_Training’ as a central node, directly linked to ‘Knowledge_Score’ and ‘Practice_Score,’ illustrating the crucial role of training in pharmacovigilance knowledge enhancement and practice. Furthermore, ‘Work_Experience_Years’ is connected to ‘Practice_Score,’ highlighting the positive impact of experience on the effectiveness of pharmacovigilance practices. The diagram also shows a link between ‘Knowledge_Score’ and ‘Public_Awareness_Score,’ suggesting the influence of healthcare professionals’ knowledge on raising public awareness. Additionally, the association of ‘Attitude_Score’ with ‘Knowledge_Score’ and ‘Practice_Score’ indicates that a positive attitude towards pharmacovigilance correlates with improved knowledge acquisition and practice implementation. This analysis visually delineates the dynamics within pharmacovigilance KAP, pointing to training and experience as key factors in enhancing pharmacovigilance capabilities among healthcare personnel.

#### 3.4.3 Network attributes of KAP variables in Yunnan Province

**Figure 7:**
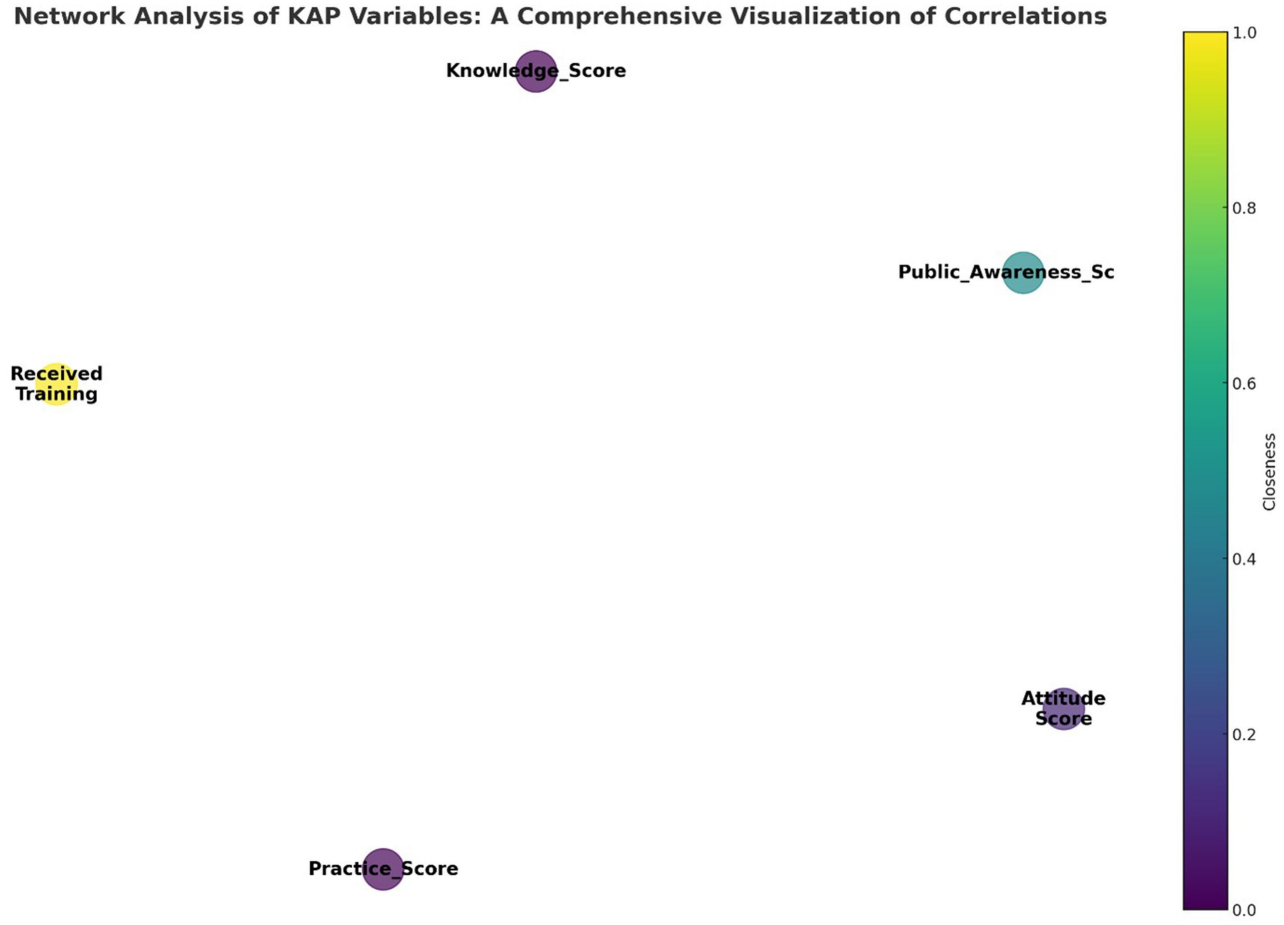
Network analysis of KAP variables: a comprehensive visualization of correlations.

Figure 7 displays a network diagram visualizing the interactions among pharmacovigilance Knowledge, Attitude, and Practice (KAP) variables within the healthcare personnel of Yunnan Province. The diagram illustrates key KAP elements as nodes, where the node size indicates the ‘Degree’ of connectivity, reflecting the number of connections to other nodes. The ‘Closeness’ of each node, shown through color intensity, reveals its centrality in the network and its potential to influence or be influenced swiftly by other nodes. ‘Received_Training’ stands out as a significant node due to its central positioning, underscoring training’s pivotal influence on KAP aspects. This underscores the potential of training as a key enhancer of pharmacovigilance practices. The diagram, along with data from Table 14, helps pinpoint effective intervention points in the network, emphasizing training’s critical role in elevating pharmacovigilance knowledge, attitudes, and thereby, practices. This color-coded network offers an analytical foundation for developing targeted pharmacovigilance enhancement strategies.

**Table 14:** Correlations among KAP variables for network analysis.

Node	Degree	Betweenness	Closeness
Knowledge_Score	4	0	0.865122
Attitude_Score	4	0	0.950356
Practice_Score	4	0	0.873815
Received_Training	4	6	1.687063
Public_Awareness_Score	4	0	1.255225

## 4 Discussion

### 4.1 Disparities in pharmacovigilance KAP among the public and healthcare personnel

The exploration of pharmacovigilance KAP within Yunnan Province disclosed notable disparities between the general public and healthcare personnel, showcasing a variegated engagement with pharmacovigilance across diverse demographic groups. Healthcare personnel were found to possess a heightened awareness of pharmacovigilance, averaging a knowledge score of 4.38 ± 3.06, a testament to their professional and educational immersion in drug safety realms. In comparison, the general public’s average knowledge score was 4.62 ± 2.70. This discrepancy, particularly the elevated awareness level of 87.6% among healthcare personnel against the public’s 57.75%, accentuates the substantial impact of professional exposure and education on pharmacovigilance understanding. It’s important to note that these findings, drawn from the study’s data, emphasize the variances in pharmacovigilance comprehension between healthcare professionals and the general population (15, 16).

Additionally, the demographic composition of the study’s participants—largely younger females and individuals already involved in healthcare professions—suggests a possible inclination of the sample towards those with pre-existing interests or a basic grasp of pharmacovigilance, potentially skewing the representativeness of the findings across Yunnan’s wider population. The significant presence of healthcare professionals (42.6%), students (23.4%), and public officials/employees (12.4%) within the sample, coupled with the observable disparity in the application of pharmacovigilance practices execution rates—75.44% in the public versus 77.5% among healthcare personnel—underscores an urgent need for targeted educational measures to narrow the knowledge-practice divide. Addressing these disparities and concerns about sample representation is crucial for future research. By striving for a more diversified participant demographic, future studies could yield a more encompassing comprehension of pharmacovigilance engagement province-wide, thereby augmenting the findings’ generalizability and relevance (17, 18).

### 4.2 Enhancing pharmacovigilance through knowledge, attitudes, and practices: insights from a comparative study

The investigation into the interdependence of KAP in pharmacovigilance underscores the foundational role of knowledge. It is identified as a critical driver shaping both attitudes and practices within this domain. The study highlights a notable disparity in pharmacovigilance engagement between the general public and healthcare professionals, emphasizing the necessity for targeted educational initiatives aimed at elevating pharmacovigilance measures. Particularly, healthcare personnel, who demonstrate higher levels of pharmacovigilance activity, present a prime focus for enhancing pharmacovigilance through improved awareness and education (19, 20).

The influence of demographic factors on pharmacovigilance KAP underscores the efficacy of demographic-specific educational strategies. Adapting outreach to cater to various demographic groups, through the use of digital tools for younger audiences and traditional methods for older ones, optimizes the impact of pharmacovigilance education. This approach not only respects the diversity of the audience but also boosts the effectiveness of pharmacovigilance initiatives. Moreover, the study points to the importance of enhancing training for healthcare personnel, suggesting a direct relationship between comprehensive pharmacovigilance education and improved patient care standards (21).

The findings advocate for a holistic strategy that encompasses knowledge dissemination, attitude development, and practice enhancement, grounded in demographic insights and tailored educational methods. This strategy addresses current pharmacovigilance KAP challenges and is pivotal for laying the foundation for continuous improvements in drug safety and healthcare delivery. By marrying these elements, the study sets forth a clear pathway towards elevating pharmacovigilance standards and achieving better health outcomes through informed, positive, and proactive pharmacovigilance practices (22).

### 4.3 Differential impact of demographic factors on pharmacovigilance KAP among healthcare personnel and the general public

The analysis of demographic characteristics and their influence on pharmacovigilance KAP in Yunnan Province presents a nuanced landscape. Among the general public, demographic variables such as age, gender, and occupation did not exhibit a significant correlation with pharmacovigilance KAP metrics. This finding diverges from existing literature and might be attributed to the unique demographic composition and methodological nuances of this study (23). The lack of significant impact from these demographic factors suggests a universal need for pharmacovigilance awareness across different segments of the public, highlighting the importance of designing education strategies that are broadly applicable and accessible to all.

In contrast, healthcare personnel demonstrated a marked distinction, showing enhanced levels of pharmacovigilance KAP. This discrepancy likely stems from their specialized training and professional exposure to pharmacovigilance concepts, underscoring the critical role of tailored educational programs aimed at healthcare providers. Such programs are essential for reinforcing drug safety practices and knowledge, suggesting an imperative for continuous professional development in pharmacovigilance (24). Moreover, the significant role of demographics (with the exception of gender) in influencing pharmacovigilance KAP among healthcare personnel underscores the potential benefits of personalized educational interventions. These interventions could be strategically designed based on age, educational background, and professional roles, to optimize pharmacovigilance outcomes (25).

This differential impact of demographic factors between the public and healthcare personnel calls for a dual approach in pharmacovigilance education. For the general public, broad-based strategies that transcend demographic barriers are recommended to ensure widespread pharmacovigilance literacy. Conversely, for healthcare personnel, more focused educational efforts that consider specific demographic characteristics may enhance the effectiveness of pharmacovigilance practices. This dual strategy not only caters to the varying needs of each group but also maximizes the potential for improving pharmacovigilance outcomes across the board.

### 4.4 Leveraging network analysis in pharmacovigilance KAP: insights and implications

Utilizing network analysis in pharmacovigilance KAP represents a significant shift towards a multifaceted examination of this domain. This method transcends conventional linear approaches by unraveling the complex interrelations and patterns that shape the pharmacovigilance field. It notably emphasizes the importance of enhancing knowledge, identifying the “Knowledge Score” as a key determinant influencing attitudes and practices. This insight underlines the critical role of knowledge in not only improving awareness but also in driving positive behavioral changes and practical applications in pharmacovigilance. Such a nuanced understanding aids in the development of targeted interventions, with the analysis pointing to the central role of “Received Training” and the beneficial effects of work experience in strengthening pharmacovigilance efforts (26).

The network analysis approach offers a comprehensive framework for identifying strategic entry points for intervention, highlighting how improvements in specific areas can generate widespread benefits across the pharmacovigilance network. By pinpointing crucial nodes like training and knowledge enhancement, it lays the groundwork for devising effective strategies aimed at bolstering pharmacovigilance practices. This perspective not only deepens our grasp of the KAP model but also charts a course for enhancing drug safety and patient care, showcasing the transformative power of network analysis in the field of pharmacovigilance (27).

### 4.5 Practical implications and limitations

This study, grounded in the KAP theoretical framework, provides a nuanced look at pharmacovigilance among Chinese healthcare personnel and the public, identifying key factors influencing its effectiveness. The findings advocate for improved dissemination of pharmacovigilance knowledge and underscore the importance of shaping positive attitudes towards drug safety (28). However, the study’s reliance on questionnaires could introduce response biases, and its focus on a single province limits the applicability of its findings across China. Future research should expand geographically to enhance the relevance and applicability of the data.

## 5 Conclusion

The study confirms a significant link between pharmacovigilance KAP among both the public and healthcare personnel in Yunnan, with demographic factors more profoundly affecting healthcare professionals. Advancing pharmacovigilance knowledge is pivotal for improving attitudes and practices, thereby raising the pharmacovigilance standard in China. Network analysis has proven invaluable in identifying key areas for intervention. Future research should broaden the scope and address this study’s limitations, including its sample size and potential for response bias, to reinforce the findings’ validity and applicability.

## Author’s note

We proposed hypotheses (*H1*–*H6* and *H1*Δ–*H6*Δ) integrating demographic elements with KAP theory, framing a holistic evaluation of pharmacovigilance KAP:

- *H1* and *H1*Δ: Knowledge positively influences attitude.
- *H2* and *H2*Δ: Attitude positively impacts practice.
- *H3* and *H3*Δ: Knowledge positively affects practice.
- *H4*–*H6* and *H4*Δ–*H6*Δ: Demographics positively correlate with knowledge, attitude, and practice, respectively.

## Data availability statement

The original contributions presented in the study are included in the article/Supplementary material, further inquiries can be directed to the corresponding authors.

## Ethics statement

The studies involving humans were approved by Biomedical Ethics Committee of Kunming Medical University, China. The studies were conducted in accordance with the local legislation and institutional requirements. Written informed consent for participation in this study was provided by the participants’ legal guardians/next of kin.

## Author contributions

DQ: Data curation, Formal analysis, Investigation, Writing – original draft. FL: Conceptualization, Project administration, Supervision, Writing – review & editing. JY: Formal analysis, Investigation, Methodology, Writing – original draft.
