# Supplementary material for: Cross-sectional study of pharmacovigilance knowledge, attitudes, and practices based on structural equation modeling and network analysis: a case study of healthcare personnel and the public in Yunnan Province
Source: Front Public Health. 2024 Mar 19;12:1358117. doi: 10.3389/fpubh.2024.1358117 (PMC10985242; doi:10.3389/fpubh.2024.1358117)
Supplement: Supplementary file 1 [file Table_1.doc]

"Survey Study on the Knowledge, Attitudes, and Practices of Healthcare Workers Regarding Pharmacovigilance - Focusing on Medical Institutions in Yunnan Province" (Survey for Healthcare Workers)

Greetings,

The Yunnan Provincial Drug Policy Research Center is conducting a research project entitled "Knowledge, Attitudes, and Practices of Healthcare Workers Regarding Pharmacovigilance - Focusing on Medical Institutions in Yunnan Province." This initiative is designed to enhance the development and optimization of pertinent policies within our region, contributing to the improvement of healthcare quality and patient safety.

Instructions for Completing the Survey:

- Please select your response by clicking on the appropriate option. All questions are formatted as multiple-choice, except where open-ended responses are specifically requested. For such items, kindly provide your answer in the allocated space.
- Rest assured, any personal information and data collected through this survey will be strictly used for the purposes of this research project. Confidentiality will be rigorously maintained in the publication of the final report. We value your honest and precise responses.

Glossary for Terms Used:

- ADR: Adverse Drug Reaction
- ADE: Adverse Drug Event
- PV: Pharmacovigilance

Your insights will significantly contribute to our understanding of pharmacovigilance practices among healthcare workers in Yunnan Province, ultimately aiding in the enhancement of drug safety and efficacy.

Thank you for your essential contribution to this study!

**I. Basic Information**

1. Your age is [Single choice] *

○A. Under 25

○B. 25-30 years

○C. 31-50 years

○D. 51-60 years

○E. Over 61 years

2. Your gender is [Single choice] *

○A. Male

○B. Female

3. Your years of service at this unit [Single choice] *

○A. 0-1 year

○B. 2-5 years

○C. 6-10 years

○D. 11-15 years

○E. Over 16 years

4. Your professional technical qualification is [Single choice] *

○A. Senior

○B. Associate Senior

○C. Intermediate

○D. Junior/Assistant

○E. Staff Level

○F. Other

5. Your highest educational level is [Single choice] *

○A. Associate degree

○B. Bachelor's degree

○C. Master's degree and above

○D. High school and vocational school

○E. Junior high school and below

6. Your position is [Single choice] *

○A. Doctor

○B. Pharmacist

○C. Nurse

○D. Technician

○E. Medical insurance department staff

7. The nature of the hospital you work in [Single choice] *

○A. Tertiary general hospital (including traditional Chinese medicine hospital)

○B. Secondary general hospital (including traditional Chinese medicine hospital)

○C. Traditional Chinese medicine specialty hospital

○D. Community health service center (including service stations)

○E. Township health centers (including health posts)

○F. Other

**II. Knowledge of Pharmacovigilance**

1. Understanding of basic pharmacovigilance concepts [Single choice] *

○A. Being vigilant about the use of drugs beyond their approved indications

○B. The science and activities related to monitoring, evaluating, understanding, and preventing adverse reactions or any other drug-related problems

○C. Harmful and unintended reactions occurring after drug use

○D. Information related to the benefit-risk profile of products

○E. I don’t know

2. Sources of pharmacovigilance knowledge and awareness (Multiple choice) [Multiple choice] *

□A. Social media platforms

□B. Television

□C. Newspapers

□D. Radio

□E. Internet

□F. School

3. Understanding of adverse drug reactions [Single choice] *

○A. Any impact of taking a drug

○B. Unexpected reactions after taking a drug

○C. Adverse reactions unrelated to the drug's intended use, occurring with proper usage and dosage of a qualified drug

○D. I don’t know

4. Have you heard of a provincial-level Adverse Drug Reaction Monitoring and Evaluation Center in Yunnan Province? [Single choice] *

○A. Yes

○B. No

5. Should female patients be asked about pregnancy when prescribing medication? [Single choice] *

○A. Yes

○B. No

1. When prescribing medication, are you aware of pharmacovigilance? [Single choice] *

○A. Yes

○B. No

2. When patients purchase medication, do you provide them with pharmacovigilance knowledge? [Single choice] *

○A. Yes

○B. No

3. Do you check the “Adverse Drug Reactions” section in the medication guide? [Single choice] *

○A. Yes

○B. No

**III. Attitudes towards Pharmacovigilance**

1. Have you had experience in conducting pharmacovigilance work? [Single choice] *

○A. Yes

○B. No

2. Measures for dealing with adverse drug reactions in pharmacovigilance work? [Multiple choice] *

□A. Notify other medical staff for consultation

□B. Stop administering the drug to the patient

□C. Take no action, as the adverse reaction will resolve on its own

□D. Treat the patient's discomfort with another drug

□E. Change the patient's medication

3. Have patients or relatives and friends ever consulted you about pharmacovigilance knowledge? [Single choice] *

○A. Often

○B. Occasionally

○C. Never

4. Preferred method for reporting adverse drug reactions in pharmacovigilance work [Single choice] *

○A. Report directly to medical professionals

○B. Phone or text message

○C. Online program designed for reporting adverse drug reactions

○D. Fill out a report form

○E. Submit an online report

○F. Do not report

5. Reasons for not reporting adverse drug reactions in pharmacovigilance work? [Multiple choice] *

□A. Unaware of the importance of reporting adverse drug reactions

□B. The adverse reaction is not very serious

□C. Don’t know how to report such reactions

□D. Unsure if the adverse reaction is related to the medication

□E. Adverse reaction resolved on its own

□F. Always report

6. Channels for collecting ADR/ADE within the hospital? [Multiple choice] *

□A. ADR monitors collect from various departments

□B. Doctors/nurses report ADR/ADE found during treatment to monitors

□C. ADR monitors collect through case studies/literature/internet and other means

□D. Doctors/nurses directly report online

□E. Other

7. Time limit for reporting new and serious ADRs within the hospital? [Single choice] *

○A. Report immediately

○B. Within 15 days

○C. Other

8. Time limit for reporting general ADRs within the hospital? [Single choice] *

○A. Within 15 days

○B. Within 30 days

○C. Other

9. Is there a regular summary and analysis of ADRs? [Single choice] *

○A. Yes

○B. No

10. Frequency of ADR summary and analysis (per year) [Single choice] *

○A. 0 times

○B. 1-2 times

○C. 3-5 times

○D. >5 times

**IV. Practices Related to Pharmacovigilance Information**

1. Are you familiar with the monitoring and reporting system for adverse drug reactions in pharmacovigilance work? [Single choice] *

○A. Very familiar

○B. Somewhat familiar

○C. Not very familiar

○D. Unfamiliar

2. Is the investigation system for fatal cases in adverse drug events in pharmacovigilance work well established? [Single choice] *

○A. Well established

○B. Fairly well established

○C. Not very well established

○D. Poorly established

3. Frequency of investigating group events of adverse drug reactions in pharmacovigilance work? [Single choice] *

○A. Once a week

○B. Once a month

○C. Once a quarter

○D. Every six months

○E. Not conducted

4. Emergency response methods for sudden adverse drug events in pharmacovigilance work? [Multiple choice] *

□A. Expert discussion system

□B. Conduct investigations, take emergency measures

□C. Clarify the nature of the event, understand the extent of the danger

□D. Report the event to higher

5. Is the management of the high-risk drug category directory in your institution well established? [Single choice] *

○A. Well established

○B. Relatively well established

○C. Not very well established

○D. Not established

6. Is the process for applying for off-label drug use well established? [Single choice] *

○A. Well established

○B. Relatively well established

○C. Not very well established

○D. Not established

7. Forms of pharmacovigilance training? [Single choice] *

○A. Self-study

○B. Internal lectures in medical institutions

○C. Specialized lectures or centralized training by provincial/municipal agencies (e.g., Drug Administration)

○D. Other

8. What does pharmacovigilance training include? (Multiple choice) [Multiple choice] *

□A. Training on pharmacovigilance-related laws, regulations, and concepts

□B. Training on the national adverse drug reaction monitoring system and China Hospital Pharmacovigilance System (CHPS)

□C. Training on standard report form filling (e.g., adverse drug reaction reports)

□D. Training on adverse drug reaction analysis/evaluation methods

□E. Reporting procedures for various monitoring events (including medication errors/risk monitoring, etc.)

□F. Other

9. Average duration of pharmacovigilance training (per session) [Single choice] *

○A. ≤0.5h

○B. 0.5-1h

○C. 1-2h

○D. >2h

**V. Judgment on Pharmacovigilance Cognition**

1. Reporting ADR is very important for patient safety [Single choice] *

○ Strongly disagree ○ Disagree ○ Neutral ○ Agree ○ Strongly agree

2. Healthcare professionals should be required to report ADR [Single choice] *

○ Strongly disagree ○ Disagree ○ Neutral ○ Agree ○ Strongly agree

3. As part of my professional practice, I do not have time to report ADR [Single choice] *

○ Strongly disagree ○ Disagree ○ Neutral ○ Agree ○ Strongly agree

4. Pharmacovigilance knowledge should be taught in undergraduate medical and pharmaceutical courses [Single choice] *

○ Strongly disagree ○ Disagree ○ Neutral ○ Agree ○ Strongly agree

5. Medical institutions or higher authorities should organize workshops or training courses to cover pharmacovigilance work [Single choice] *

○ Strongly disagree ○ Disagree ○ Neutral ○ Agree ○ Strongly agree

6. I have a professional duty to report ADR [Single choice] *

○ Strongly disagree ○ Disagree ○ Neutral ○ Agree ○ Strongly agree

7. I am concerned that reporting ADR to higher authorities may have legal consequences [Single choice] *

○ Strongly disagree ○ Disagree ○ Neutral ○ Agree ○ Strongly agree

8. I would be encouraged to report more ADRs if there were compensation [Single choice] *

○ Strongly disagree ○ Disagree ○ Neutral ○ Agree ○ Strongly agree

9. I would be encouraged to report more ADRs if patient information were automatically filled from HIS software into the prepared ADR report [Single choice] *

○ Strongly disagree ○ Disagree ○ Neutral ○ Agree ○ Strongly agree

10. I would be encouraged to report more ADRs if provided with general education on the importance of pharmacovigilance [Single choice] *

○ Strongly disagree ○ Disagree ○ Neutral ○ Agree ○ Strongly agree

Thank you again for your careful completion of this survey!
