# Supplementary material for: Cross-sectional study of pharmacovigilance knowledge, attitudes, and practices based on structural equation modeling and network analysis: a case study of healthcare personnel and the public in Yunnan Province
Source: Front Public Health. 2024 Mar 19;12:1358117. doi: 10.3389/fpubh.2024.1358117 (PMC10985242; doi:10.3389/fpubh.2024.1358117)
Supplement: Supplementary file 2 [file Table_2.doc]

"Survey Study on the Knowledge, Attitudes, and Practices of Patients/Public Regarding Pharmacovigilance - Focusing on Medical Institutions in Yunnan Province" (Survey for Patients/Public)

Greetings,

The Yunnan Provincial Drug Policy Research Center has initiated a research project titled "Knowledge, Attitudes, and Practices of Patients/General Public Regarding Pharmacovigilance - Focusing on Medical Institutions in Yunnan Province." This endeavor is designed to bolster the formulation and refinement of related policies within our province, aiming at enhancing public health outcomes.

Instructions for Survey Participation:

- Please select your answers by clicking the appropriate option. Unless otherwise indicated, all questions are multiple-choice. For open-ended questions, kindly type your response in the provided space.
- We assure you that all personal information and data collected in this survey will be exclusively used for this research project. Confidentiality will be strictly maintained in the final report. Your honest and accurate responses are greatly appreciated.

Terminology Guide:

- ADR: Adverse Drug Reaction
- ADE: Adverse Drug Event
- PV: Pharmacovigilance

Your participation plays a crucial role in advancing our understanding of pharmacovigilance and its impact on patient care and safety in Yunnan Province.

Thank you for your valuable contribution!

**I. Basic Information**

1. Your age is [Single choice] *

○A. Under 18

○B. 18-30 years

○C. 31-50 years

○D. 51-60 years

○E. Over 61 years

2. Your gender is [Single choice] *

○A. Male

○B. Female

3. Your occupation is? [Single choice] *

○A. Farmer

○B. Worker

○C. Businessperson

○D. Government or institution employee

○E. Medical staff

○F. Student

○G. Teacher

○H. Self-employed

○I. Police officer

○J. Driver

○K. Military personnel

○L. Other

4. What is your usual channel for purchasing medications? [Single choice] *

○A. Hospital

○B. Community health service center

○C. Retail pharmacy

○D. Online pharmacy

5. Do you have any chronic diseases? [Single choice] *

○ Heart disease

○ Hypertension

○ Diabetes

○ Rheumatism

○ Other

○ None

6. Do you have family members or friends working in the pharmacovigilance field? [Single choice] *

○A. Yes

○B. No

**II. Knowledge about Pharmacovigilance**

1. Understanding of basic pharmacovigilance concepts [Single choice] *

○A. Being vigilant about the use of drugs beyond their approved indications

○B. The science and activities related to monitoring, evaluating, understanding, and preventing adverse reactions or any other drug-related problems

○C. Harmful and unintended reactions occurring after drug use

○D. Information related to the benefit-risk profile of products

○E. I don’t know

2. Sources of pharmacovigilance knowledge* [Multiple choice] *

□A. Social media platforms

□B. Television

□C. Newspapers

□D. Radio

□E. Internet

□F. School

□G. Never heard of it

3. Adverse drug reactions that you think may require hospitalization? [Single choice] *

○A. Life-threatening reactions

○B. Reactions requiring another drug for treatment

○C. Reactions that resolve on their own

○D. I don’t know

4. Understanding of adverse drug reactions [Single choice] *

○A. Any impact of taking a drug

○B. Unexpected reactions after taking a drug

○C. Adverse reactions unrelated to the drug's intended use, occurring with proper usage and dosage of a qualified drug

○D. I don’t know

5. How long have you been aware of pharmacovigilance knowledge? [Single choice] *

○A. Within 1 year

○B. 1-5 years

○C. 6-10 years

○D. More than 10 years

○E. Never understood

6. Do you worry about adverse drug reactions when taking medications? [Single choice] *

○A. Yes

○B. No

7. When purchasing medication, do you consult doctors or pharmacists about pharmacovigilance? [Single choice] *

○A. Yes

○B. No

8. Do you check the “Adverse Drug Reactions” section in the medication guide? [Single choice] *

○A. Yes

○B. No

**III. Attitudes towards Pharmacovigilance**

1. Have you ever attended a lecture on pharmacovigilance? [Single choice] *

○A. Yes

○B. No

2. What do you think are the measures for dealing with adverse drug reactions? [Multiple choice] *

□A. Notify medical staff

□B. Stop taking the drug

□C. Take no action, as the adverse reaction is tolerable

□D. Take no action, as the adverse reaction will resolve on its own

□E. Use another drug to treat discomfort

□F. Switch to another drug

3. Have you ever consulted relatives, friends, doctors, or pharmacists about pharmacovigilance knowledge? [Single choice] *

○A. Often

○B. Occasionally

○C. Never

4. Sources of pharmacovigilance knowledge during medical consultation? [Multiple choice] *

□A. Drug promotional materials

□B. Pharmacists

□C. Doctors

□D. Internet

□E. Nurses

□F. Relatives, friends

5. If you encounter an adverse drug reaction, what will you do? [Multiple choice] *

□A. Report directly to medical professionals

□B. Search or consult online

□C. Use online programs designed for reporting adverse drug reactions

□D. Fill out a report form

□E. Submit an online report

□F. Not report

6. Reasons for not reporting observable adverse drug reactions to the doctor in daily life? [Multiple choice] *

□A. Unaware of the importance of reporting adverse drug reactions

□B. The adverse reaction is not very serious

□C. Don’t know how to report such reactions

□D. Unsure if the adverse reaction is related to the medication

□E. Adverse reaction resolved on its own

□F. Always report

**IV. Practices Related to Pharmacovigilance**

1. When taking medications daily, are you aware of monitoring for pharmacovigilance? [Single choice] *

○A. Yes

○B. Occasionally

○C. Not really

○D. No

2. When taking medications daily, are you aware of preventing issues in pharmacovigilance? [Single choice] *

○A. Yes

○B. Occasionally

○C. Not really

○D. No

3. Do you actively seek to understand the pharmacovigilance knowledge of medications you take daily? [Single choice] *

○A. Once a week

○B. Once a month

○C. Once a quarter

○D. Once every six months

○E. Do not actively seek to understand

4. Do you read the pharmacovigilance information in the medication guide before taking medication? [Single choice] *

○A. Every time

○B. Occasionally

○C. Not often

○D. Never

5. How do you learn about pharmacovigilance knowledge? [Single choice] *

○A. Self-study

○B. Internal lectures in medical institutions

○C. Provincial/municipal agencies (e.g., drug administration) conducting specialized lectures or centralized training

○D. Other

○E. Never learn

**V. Judgment on Pharmacovigilance-Related Situations**

1. Collecting pharmacovigilance information can improve medication safety. [Single choice] *

○ Strongly disagree ○ Disagree ○ Neutral ○ Agree ○ Strongly agree

2. Doctors and pharmacists should inform consumers (patients) about pharmacovigilance knowledge (adverse drug reactions). [Single choice] *

○ Strongly disagree ○ Disagree ○ Neutral ○ Agree ○ Strongly agree

3. Government departments should mandate medical staff to report patients' adverse drug reactions. [Single choice] *

○ Strongly disagree ○ Disagree ○ Neutral ○ Agree ○ Strongly agree

4. Government departments should enhance openness and increase publicity for direct patient reporting channels for adverse drug reactions. [Single choice] *

○ Strongly disagree ○ Disagree ○ Neutral ○ Agree ○ Strongly agree

5. Government departments should penalize medical institutions for failing to legally report adverse drug reactions. [Single choice] *

○ Strongly disagree ○ Disagree ○ Neutral ○ Agree ○ Strongly agree

6. To improve the ability of medical staff in pharmacovigilance, medical institutions should conduct regular training. [Single choice] *

○ Strongly disagree ○ Disagree ○ Neutral ○ Agree ○ Strongly agree

7. Government agencies and medical institutions should organize public activities or lectures, highlighting the importance of pharmacovigilance work. [Single choice] *

○ Strongly disagree ○ Disagree ○ Neutral ○ Agree ○ Strongly agree

Thank you again for your careful completion of this survey.
